# Forced rather than voluntary exercise entrains peripheral clocks via a corticosterone/noradrenaline increase in PER2::LUC mice

**DOI:** 10.1038/srep27607

**Published:** 2016-06-08

**Authors:** Hiroyuki Sasaki, Yuta Hattori, Yuko Ikeda, Mayo Kamagata, Shiho Iwami, Shinnosuke Yasuda, Yu Tahara, Shigenobu Shibata

**Affiliations:** 1 Laboratory of Physiology and Pharmacology, School of Advanced Science and Engineering, Waseda University, Shinjuku-ku, Tokyo 162-8480, Japan

## Abstract

Exercise during the inactive period can entrain locomotor activity and peripheral circadian clock rhythm in mice; however, mechanisms underlying this entrainment are yet to be elucidated. Here, we showed that the bioluminescence rhythm of peripheral clocks in PER2::LUC mice was strongly entrained by forced treadmill and forced wheel-running exercise rather than by voluntary wheel-running exercise at middle time during the inactivity period. Exercise-induced entrainment was accompanied by increased levels of serum corticosterone and norepinephrine in peripheral tissues, similar to the physical stress-induced response. Adrenalectomy with norepinephrine receptor blockers completely blocked the treadmill exercise-induced entrainment. The entrainment of the peripheral clock by exercise is independent of the suprachiasmatic nucleus clock, the main oscillator in mammals. The present results suggest that the response of forced exercise, but not voluntary exercise, may be similar to that of stress, and possesses the entrainment ability of peripheral clocks through the activation of the adrenal gland and the sympathetic nervous system.

The mammalian circadian system consists of three major components, including controlling the input, oscillation, and the output pathways. The central “master” pacemaker is located in the suprachiasmatic nucleus (SCN) of the hypothalamus, with numerous extra-SCN oscillators distributed throughout the brain and peripheral organs. Output pathways bring about changes in the temporal organization of animal physiology, endocrinology, and behavior^1^,^2^. The SCN-driven locomotor activity rhythm is entrained not only by photic stimulation, such as the LD cycle, but also by non-photic stimuli, including increases in locomotor activity and/or arousal as a result of voluntary or forced scheduled exercise^2^,^3^,^4^,^5^,^6^,^7^,^8^,^9^,^10^,^11^. The cellular and molecular mechanisms of photic entrainment in the SCN have been extensively investigated (see review 11). At the molecular level, a feedback loop of transcriptional activation by CLOCK/BMAL1 and repression by PER/CRY complexes drives the rhythmic expression of these proteins in each cell over a ~24-h cycle. In addition, *Dbp* and *Rev-erbα*, which encode a transcriptional activator and repressor, respectively, also show oscillatory expression over periods of approximately 24 h and regulate clock-controlled output genes^1^,^2^. Expression of *Per1* and *Per2*, and their proteins was induced in the SCN by light pulses delivered at the appropriate nighttime phase^12^,^13^,^14^. Photic signals induce *Per1* expression through the activation of second messenger cascades, including ERK, PKA, and Ca2+/calmodulin-dependent protein kinase II (CaMKII), which ultimately leads to the transcriptional activation of *Per1* genes via the activation of cAMP response elements in their promoters^15^,^16^,^17^. Non-photic stimuli induced by both, novel wheel confinement and benzodiazepine drugs, can suppress the expression of *Per1* and *Per2* mRNA in the SCN. This can, at times, result in a behavioral phase shift^18^,^19^. Non-photic stimuli suppress ERK phosphorylation in the SCN^20^, thus, activation and suppression of the ERK pathway may be involved in mediating responses to both, photic and non-photic signaling.

In addition to the centrally located SCN-driven system, the peripheral clock-driven system is also known to play an important role in circadian rhythms. Rodent peripheral clock oscillation is entrained by a restricted feeding schedule^21^,^22^ and also by scheduled exercise^23^,^24^,^25^. Recent reports have demonstrated that insulin release is a critical step in the entrainment of the mouse peripheral clock by restricted feeding^26^,^27^. Thus, insulin acts as an entrainment signal^26^,^28^,^29^. However, the mechanisms of entrainment of the peripheral clock by scheduled exercise are not well understood.

In the present study, two different exercise protocols were used; voluntary wheel-running exercise and forced treadmill exercise. Wheel-running activity in mice is not only a source of exercise but also provides self-motivation, and it is considered to be a reward behavior^30^. Previous studies have demonstrated that wheel-running activity plays an important role in modulating circadian rhythms in rodents, whereas forced treadmill exercise does not^31^,^32^. Thus, it has been hypothesized that wheel-running activity may be a motivational state that affects circadian rhythmicity. Therefore, in the current experiment, we examined the effects of voluntary or forced exercise on the entrainment of peripheral organ clocks. To confirm our working hypothesis, forced wheel-running exercise instead of voluntary wheel-running was also included.

The hypothalamic-pituitary-adrenal (HPA) axis and sympathetic nervous system are the main neuroendocrine pathways that are activated in response to stressful stimuli and physical challenges, such as exercise. The peripheral organ clock is entrained by the daily administration of glucocorticoids^33^,^34^,^35^,^36^ and sympathetic agonists^36^,^37^, as well as by restraint or social-defeat^36^. In the present experiments using mice, we examined whether exercise-induced phase shift could be blocked by adrenalectomy and/or treatment with adrenergic receptor blockers. The *in vivo* bioluminescence imaging system (IVIS), with PER2::LUC mouse^26^, was used to assess the phase and amplitude of peripheral clock gene expression *in vivo*.

## Results

### Scheduled forced treadmill or voluntary wheel exercise shifts the circadian PER2::LUC bioluminescence rhythm in peripheral tissues

**Figure 1:**
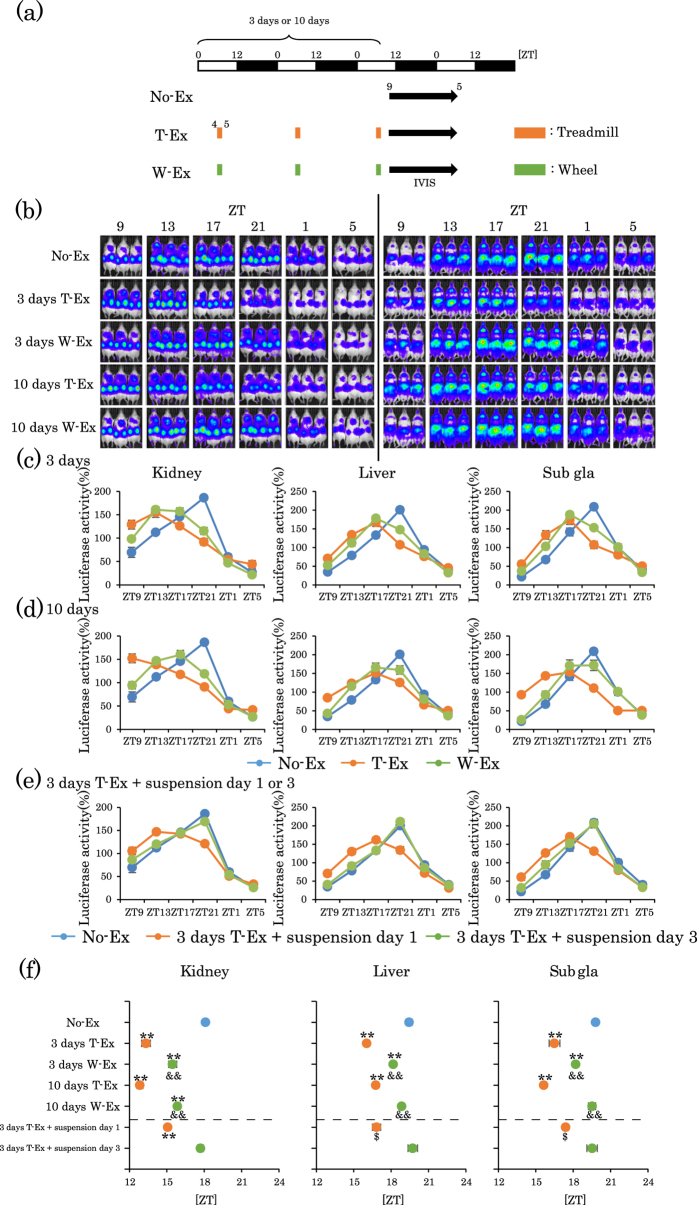
Treadmill or wheel-running exercise during the inactive period produces phase advance of mouse peripheral clocks. (**a**) Experimental schedule. The white and black bars indicate environmental 12 h light and dark conditions. The orange short bar indicates forced exercise periods. The green short bar indicates voluntary exercise periods. The black arrow indicates the period of measurement, using IVIS. (**b**) Representative images of *in vivo* PER2::LUC bioluminescence in the kidney (left panels) and in the liver and submandibular gland (right panels). (**c**,**d**) Comparison of PER2::LUC expression rhythms of peripheral clocks in mice after non-exercise (No-Ex), treadmill-Ex (T-Ex) or wheel-running exercise (W-Ex). Exercise was applied for 3 days (**c**) or 10 days (**d**). (**e**) PER2::LUC expression rhythms of peripheral clocks in mice on day 1 or 3 of exercise suspension after 3 consecutive days treadmill. (**f**) Average peak phase of PER2::LUC rhythms in each tissue after exercise treatment indicated in (**c**–**e**). All the values are represented as mean ± SEM (No-Ex, n = 8; 3 days T-Ex, n = 6; 3 days W-Ex, n = 6; 10 days T-Ex, n = 6; 10 days W-Ex, n = 6; 3 days T-Ex + suspension day 1, n = 6; 3 days T-Ex + suspension day 3, n = 6). **p < 0.01 versus No-Ex, evaluated using the one-way ANOVA test with the Tukey *post-hoc* test. ^&&^p < 0.01 versus T-Ex at the same period and span, evaluated using the one-way ANOVA test with Tukey *post-hoc* test. ^$^p < 0.05 versus No-Ex evaluated using the Kruskal-Wallis test with Dunn *post-hoc* test. No exercise, treadmill exercise or wheel-running exercise: No-Ex, T-Ex or W-Ex, respectively.

There have been several reports indicating that forced exercise during the inactive period shifts the circadian clock in peripheral tissues^23^,^24^,^25^. To check whether our experimental system concurs with previous reports, mice were forced to exercise during the inactivity period. PER2::LUC mice were divided into two groups: the non-exercise group (No-Ex) and the treadmill exercise group (T-Ex). Mice were forced to exercise on the treadmill during inactivity period at Zeitgeber time (ZT, ZT0 is lights-on and ZT12 is lights-off time) four to five times per day for three consecutive days. After scheduled exercise, we measured the bioluminescence rhythms of the kidney, liver, and submandibular glands (Sub gla) (Fig. 1a). In Fig. 1b, the raw PER2::LUC bioluminescence images of measured kidney (left panel) and liver/Sub gla (right panel) are shown representative of the circadian temporal order to show the phase-shifts. The phases of the bioluminescence rhythms of the peripheral clocks in the T-Ex group were significantly phase advanced compared to those in the No-Ex group (Fig. 1c). When the duration of forced treadmill exercise was set as 0.5, 1, and 2 h, the 1 and 2 h groups showed similar values of phase advances (Supplementary Fig. S1a,b). We also compared the two groups with different running speeds, constant speed versus gradually increased speed. The former is a constant speed of 12 m/min for 60 min, and the latter is a speed of 9 m/min for 20 min, 12 m/min for 20 min, and 15 m/min for 20 min. The phase advance values were comparable between the two groups (Supplementary Fig. S1c,d). From these data, the speed of forced treadmill exercise was fixed at 12 m/min for 60 min for the next experiments. Although treadmill exercise affected the phase of peripheral clocks, this exercise did not affect the rhythmicity and amplitude of oscillation of the peripheral clocks assessed by cosinor fitting analysis (Supplementary Table S1).

In the next experiment, we compared the effect of forced treadmill exercise to that of voluntary wheel-running exercise. The running distance and speed of running were slightly higher in the wheel group than in the treadmill groups after approximately Day four of daily exercise conducted at ZT4–5 or at ZT20–21 (Supplementary Fig. S2a,c). In addition, increases in body temperature between forced exercise and voluntary exercise groups were similar (Supplementary Fig S2d,e). Under similar exercise conditions, the values of phase advances were significantly smaller in the wheel-running group compared to the forced-running group (Fig. 1f).

As non-photic stimulation at late time points during the active period produces a phase delay, in the current experiment, treadmill or wheel exercise was applied for 60 min at ZT20–21. Treadmill exercise produced a larger phase delay of peripheral clocks when compared to wheel-running exercise (Supplementary Fig. S3).

In the next experiment, we examined whether treadmill exercise-induced phase advances and phase delays exhibit a tolerance effect. Treadmill exercise for 10 days continued to produce phase advances or phase delays (Fig. 1b,d,f and Supplementary Fig. S3b,S3d and S3e), whereas, 10 days of wheel-running exercise conferred a tolerance effect on both phase advance and phase delay (Fig. 1b,d,f and Supplementary Fig. S3b,S3d and S3e). If the three days of exercise had caused a phase clock oscillation, phase shift should be maintained for several cycles. Therefore, in the next experiment, we examined whether treadmill exercise-induced phase advances returned to normal after one and three days of exercise suspension (following three days of exercise). The phase remained advanced after one day of suspension in T-ex mice compared to that of No-Ex mice (Fig. 1e,f). However, after three days of suspension, phase advance was returned to control levels (Fig. 1e,f). These results suggest that forced treadmill exercise, rather than voluntary wheel-running exercise, produced a phase advance and a phase delay in peripheral clocks during the inactive and active periods, respectively.

### Scheduled forced treadmill or voluntary wheel exercise shifts mRNA expression rhythms of clock genes in various peripheral tissues

To confirm the phase shift in PER2::LUC rhythm, the expression of other clock genes was examined in various peripheral organs including the lung, skeletal muscles, and adrenal gland (Supplementary Fig. S4 and Table S2). Similar to PER2::LUC rhythm, *Per2* and *Bmal1* mRNA expression rhythms were advanced in all organs (especially, *Per2* in the liver and lung; *Bmal1* in the kidney and lung) after exercise at ZT4–5, and these values were relatively larger in T-Ex mice organs compared with those of wheel exercise (W-Ex) mice organs (especially, *Per2* in the kidney and sub gla; *Bmal1* in the kidney and lung) (Supplementary Table S2). Whereas, exercise-induced phase advances in *Per1* and *Rev-erbα* mRNA expression rhythms were comparable between T-Ex and W-Ex groups, excluding T-Ex induced large advance of *Per1* and *Rev-erbα* mRNA expression rhythms in the kidney and adrenal, respectively. In the gastrocnemius muscle, values of phase advance of all clock gene mRNA expression were almost same between T-Ex and W-Ex groups (Supplementary Fig. S4 and Table S2). When expression in peripheral organs was compared, there was no clear organ dependency of phase shift after exercise. In the next experiment, we examined whether the daily rhythm of serum corticosterone levels was shifted by three days of treadmill exercise or wheel-running exercise (Supplementary Fig. S4a). The daily rhythm of serum corticosterone levels was also advanced by 2 h after three days of treadmill and wheel exercise (Supplementary Fig. S4c and Supplementary Table S2). To elucidate the effects of exercise-induced phase shift on expression of clock genes as well as clock-controlled steroidogenic acute regulatory protein (*StAR*; the rate-limiting step of glucocorticoid biosynthesis)^38^,^39^, the daily rhythm of *StAR* was examined. Both treadmill and wheel exercise did not affected *StAR* expression rhythm in the adrenal gland (Supplementary Fig. S4d, Table S2). These results suggest that forced treadmill exercise, rather than voluntary wheel-running exercise, produced phase advances in the mRNA expression rhythm of various clock genes in various peripheral tissues.

### Scheduled forced wheel-running exercise shifts the circadian clocks in peripheral tissues

**Figure 2:**
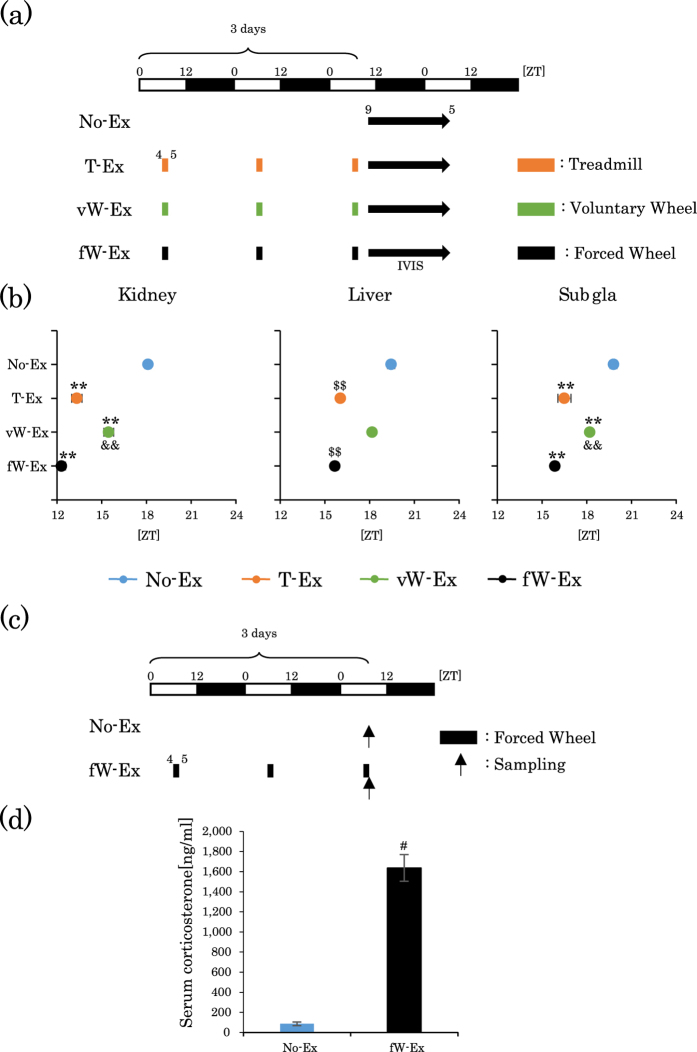
Forced wheel-running exercise during the inactive period produces phase advance and corticosterone release. (**a**) Experimental schedule. The white and black bars indicate environmental 12 h light and dark conditions. The orange short bar indicates forced treadmill exercise periods. The green short bar indicates voluntary wheel exercise period. The black short bar indicates forced wheel-running exercise periods (ZT4–5.5, for 90 min). The black arrow indicates the period of measurement using IVIS. (**b**) Average peak phase of PER2::LUC rhythms in each tissue after exercise. (**c**) Experimental schedule. The white and black bars indicate environmental 12 h light and dark conditions. The black short bar indicates forced wheel-running exercise periods (ZT4–5.5, for 90 min). The black arrow indicates the sampling time. (**d**) Serum corticosterone levels after 3 days of forced wheel-running exercise. All values are represented as mean ± SEM (No-Ex, n = 8; T-Ex, n = 6; vW-Ex, n = 6; fW-Ex, n = 6). **p < 0.01 versus No-Ex, ^&&^p < 0.01 versus fW-Ex, evaluated using the one-way ANOVA test with Tukey *post-hoc* test. ^$$^p < 0.01 versus No-Ex evaluated using the Kruskal-Wallis test with Dunn *post-hoc* test. ^#^p < 0.05 versus No-Ex evaluated using the Mann-Whitney test. No exercise, treadmill, voluntary wheel or forced wheel exercise: No-Ex, T-Ex, vW, or fW respectively.

**Figure 3:**
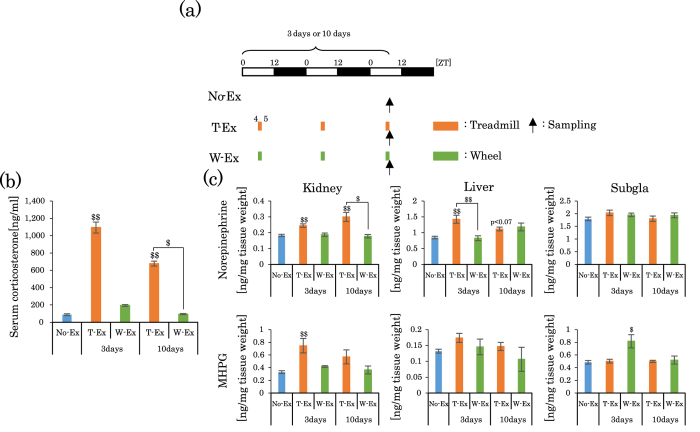
Treadmill or wheel-running exercise during the inactive period increases the levels of serum corticosterone and peripheral tissue NE/MHPG. (**a**) Experimental schedule. The white and black bars indicate environmental 12 h light and dark conditions. The orange short bar indicates forced exercise periods. The green short bar indicates voluntary exercise periods. The black arrow indicates the sampling time. (**b**) Serum corticosterone level after 3 days or 10 days of treadmill exercise or wheel-running exercise. (**c**) NE and MHPG levels in the kidney, liver and Sub gla after 3 days or 10 days of treadmill exercise or wheel-running exercise. All values are represented as mean ± SEM (No-Ex, n = 13; 3 days T-Ex, n = 4; 3 days W-Ex, n = 6; 10 days T-Ex, n = 5; 10 days W-Ex, n = 6). ^$$^p < 0.01 ^$^p < 0.05 versus No-Ex, evaluated using the Kruskal-Wallis test with Dunn *post-hoc* test. No exercise, treadmill exercise or wheel-running exercise: No-Ex, T-Ex, or W-Ex, respectively.

Voluntary wheel-running may be a motivational and less stressful exercise compared to forced exercise^30^. To clarify our hypothesis that differences in the forced or voluntary exercise, but not those in the type of running exercise, can shift the peripheral clocks, we examined whether forced wheel-running exercise, instead of voluntary wheel-running, produces a big-phase advance of the peripheral clock. Mice could not be tracked at a wheel-running speed of 12 m/min; therefore, the speed of forced wheel-running was set as 8 m/min. Forced wheel-running exercise (8 m/min for 1.5 h) produced phase advances as well as corticosterone release, which were comparable to forced treadmill exercise (12 m/min for 1 h), but not to voluntary wheel-running (approximately 12 m/min for 1 h) (Figs 2 and 3b). These results suggest that forced rather than voluntary exercise can entrain peripheral clocks.

### Increases in corticosterone and norepinephrine (NE) levels after treadmill or wheel-running exercise

As exercise is believed to be a kind of stress, in the next experiment, we examined whether the adrenal gland and sympathetic adrenergic activation are involved in treadmill exercise-induced phase advances. To assess sympathetic nervous activity, we evaluated the levels of NE and its metabolite 3-Methoxy-4-hydroxyphenylglycol (MHPG) in peripheral tissues. For treadmill exercise, but not wheel-running exercise, ZT4–5 significantly increased not only serum corticosterone levels (Fig. 3a,b) but also NE/MHPG levels in the kidney and liver (Fig. 3c), compared to the No-Ex groups. When then 3 days and 10 days exercise groups were compared, corticosterone levels were relatively dampened in the 10 days group (Fig. 3b). Exercise-induced increases in the levels of corticosterone and NE/MHPG were also examined in the treadmill groups during ZT20–21. Similar to the ZT4–5 groups, corticosterone levels were strongly increased by treadmill exercise, but not by wheel-running exercise (Supplementary Fig. S5). In contrast, the levels of NE/MHPG, during the response to treadmill or wheel exercise, varied among peripheral organs (Supplementary Fig. S5). These results suggest that increases in the levels of corticosterone and NE may be related to the exercise-induced phase shifts of peripheral clocks.

The serum corticosterone levels at ZT5 increased after three consecutive days of treadmill exercise (Fig. 3b); however, the serum corticosterone levels at ZT5 returned to control levels after one and three days of exercise suspension (after 3 days treadmill exercise) (No-Ex, 87.1 ± 9.9 ng/ml; 3 days T-Ex, 1093 ± 62.8; 1 day suspension in T-Ex, 66.7 ± 29.2; 3 day suspension, 82.6 ± 24.4). These results suggest that transient increases in the levels of corticosterone may be related to the exercise-induced phase shifts of peripheral clocks. To examine the involvement of adrenocorticotropic hormone (ACTH) in corticosterone release, we simultaneously measured serum ACTH and corticosterone levels after 0.5 and 1 h of exercise. Both treadmill and wheel-running exercise equally increased ACTH levels (Supplementary Fig. S6b). In contrast, treadmill exercise induced a marked increase in corticosterone levels compared to those induced by wheel-running exercise (Supplementary Fig. S6c, Fig. 3).

### Effect of adrenalectomy (ADX) and adrenergic receptor blockers on phase advances induced by treadmill exercise

**Figure 4:**
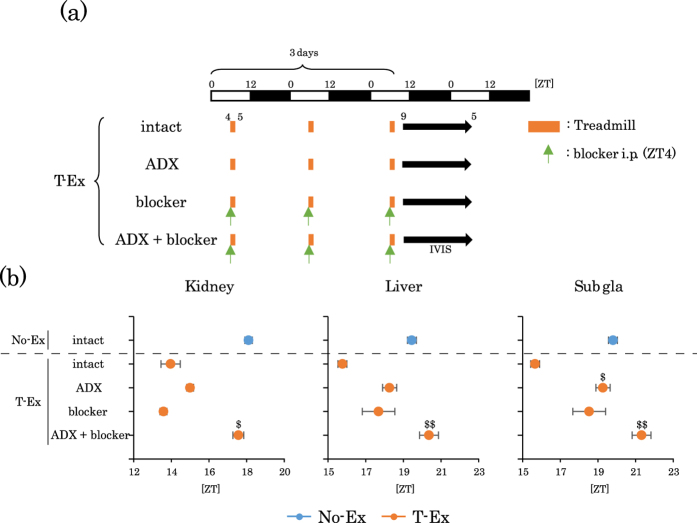
Effect of adrenalectomy and/or adrenergic receptor blockers on the treadmill exercise-induced phase advance of peripheral clocks. (**a**) Experimental schedule. The white and black bars indicate environmental 12 h light and dark conditions. The orange short bar indicates exercise periods. The black arrow indicates the periods of measurement using IVIS. The green arrow indicates the injection time of the blocker. ADX, adrenalectomized mice. Blockers, prazosin hydrochloride (1 mg/kg i.p.) and propranolol hydrochloride (2 mg/kg) were administered 30 min before exercise for 3 consecutive days. (**b**) The average peak phase of PER2::LUC rhythms in each tissue under different experimental conditions. All values are represented as mean ± SEM (No-Ex intact, n = 8; T-Ex intact, n = 6; T-Ex ADX, n = 6; T-Ex Blocker, n = 6; T-Ex ADX + Blocker, n = 5). ^$$^p < 0.01 ^$^p < 0.05 versus T-Ex intact, evaluated using the Kruskal-Wallis test with Dunn *post-hoc* test. No exercise or treadmill exercise: No-Ex or T-Ex, respectively.

**Figure 5:**
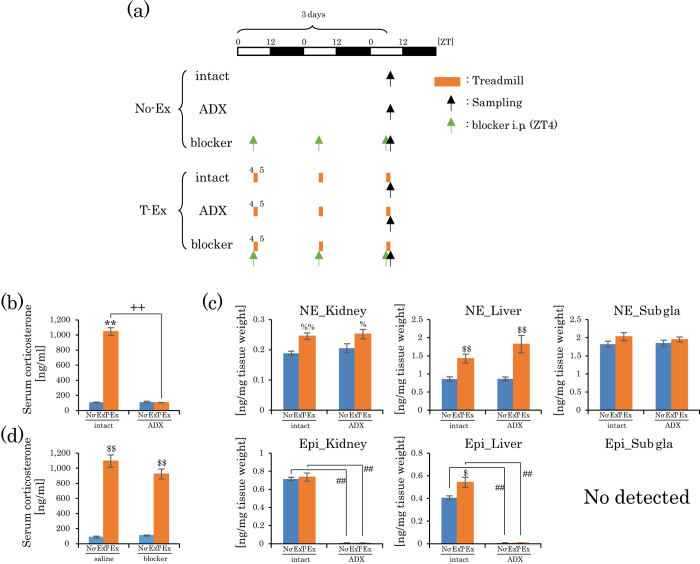
Increase in NE levels by treadmill exercise in ADX mice and increase in corticosterone levels by treadmill exercise in adrenergic blocker-treated mice. (**a**) Experimental schedule. The white and black bars indicate environmental 12 h light and dark conditions. The orange short bar indicates exercise periods. The black and green arrows indicate the sampling time and blocker injection time, respectively. (**b**) Serum corticosterone level after 3 days of treadmill exercise in intact and ADX mice. Data (**b**) are represented as mean ± SEM (intact No-Ex, n = 4; intact T-Ex, n = 4; ADX No-Ex, n = 7; ADX T-Ex, n = 4). (**c**) NE and epinephrine levels in the kidney, liver and Sub gla after 3 days of treadmill exercise in intact and ADX mice. Data (**c**) is presented as mean ± SEM (intact No-Ex, n = 8; intact T-Ex, n = 8; ADX No-Ex, n = 7; ADX T-Ex, n = 9). (**d**) Serum corticosterone levels after 3 days of treadmill exercise in intact saline or adrenergic receptor blocker-treated mice. Data (**d**) is represented as mean ± SEM (saline No-Ex, n = 6; saline T-Ex, n = 6; blocker No-Ex, n = 6; blocker T-Ex, n = 6). **p < 0.01 versus No-Ex at same condition (intact or ADX), evaluated using the two-way ANOVA test with Tukey *post-hoc* test.^++^p < 0.01 versus intact at the same exercise condition (No-Ex or T-Ex), evaluated using the two-way ANOVA test with Tukey *post-hoc* test. ^%^p < 0.01 ^%^p < 0.05 versus No-Ex under the same conditions (intact or ADX), evaluated using the two-way ANOVA test with Sidak *post-hoc* test. ^$$^p < 0.01 ^$^p < 0.05 versus No-Ex at same condition (intact or ADX), evaluated using the Mann-Whitney test. ^##^p < 0.01 versus intact under the same exercise condition (No-Ex or T-Ex), evaluated using the Mann-Whitney test. No exercise or treadmill exercise: No-Ex or T-Ex, respectively.

We examined if ADX and adrenergic receptor blockers could inhibit exercise-induced phase advances. ADX and/or adrenergic receptor blockers minimally affected the phase of the peripheral clocks under non-exercise conditions (Supplementary Fig. S7). Phase advance was significantly blocked by ADX in the sub gla clock, and was moderately blocked by either ADX or adrenergic receptor blockers in the kidney and liver clocks; in addition, phase advance was completely blocked by ADX plus adrenergic receptor blockers in all organ clocks (Fig. 4a,b). When serum corticosterone levels and NE levels were examined after treadmill exercise in ADX mice, the corticosterone levels were clearly dampened in these ADX mice; however, the NE levels were still significantly increased in the kidney and liver (Fig. 5b,c). When corticosterone levels were examined after treadmill exercise under blockade of the adrenergic receptors, serum corticosterone levels were still significantly increased (Fig. 5d). These results could explain why only ADX plus adrenergic receptor blockers could produce a complete blockade of the treadmill exercise-induced phase advances, whereas, the individual blockade of corticosterone and adrenergic stimulation produced only a partial blockade of the phase advance of the peripheral clocks in kidney and liver tissues (Figs 4a,b and 5), but not in sub gla. Overall, the present results suggest that forced exercise provided large phase shifts in peripheral clocks through the activation of both glucocorticoids and the sympathetic nervous system.

### Role of SCN on forced treadmill exercise-induced shifts of the circadian clocks in peripheral tissues

**Figure 6:**
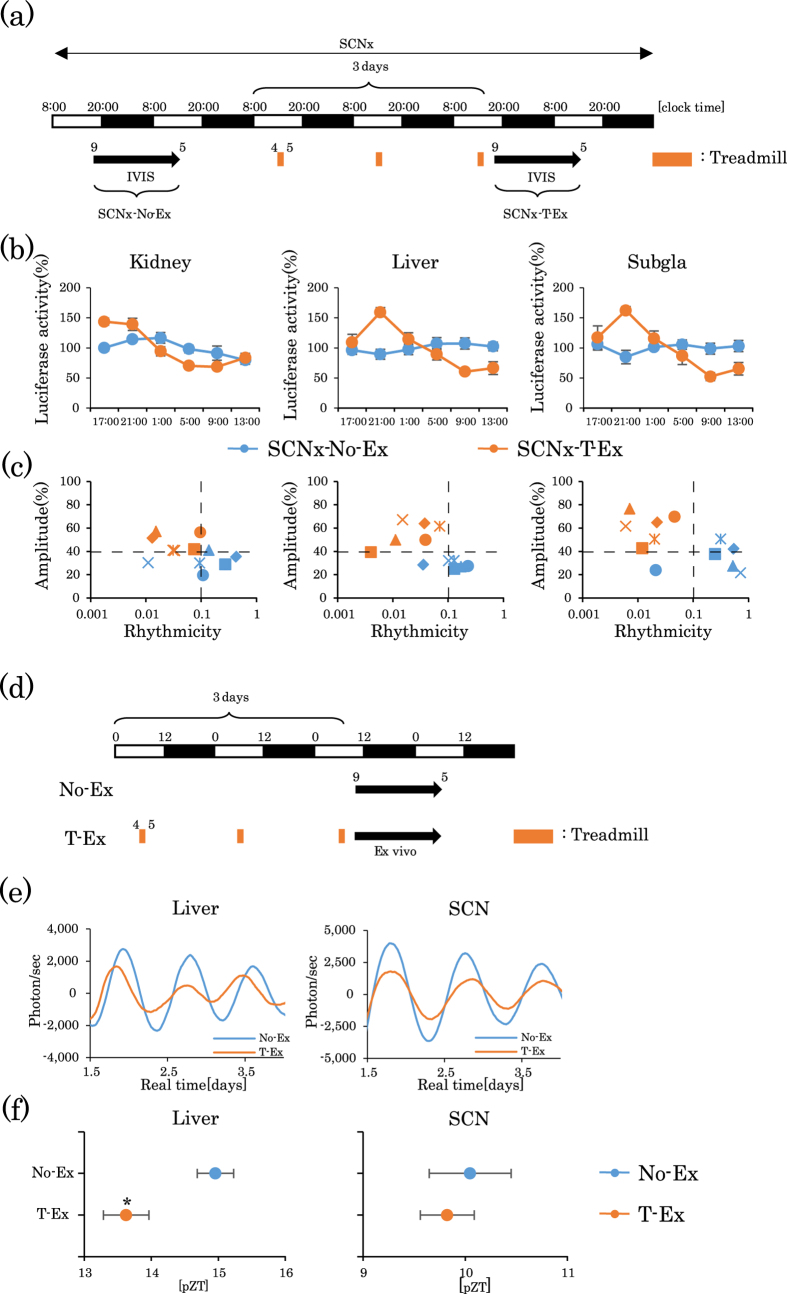
Lack of involvement of the suprachiasmatic nucleus (SCN) in the treadmill exercise-induced phase shift of mouse peripheral clocks. (**a**) Experimental schedule. The white and black bars indicate environmental 12 h light and dark conditions. The orange short bar indicates exercise periods. The black arrow indicates the period of measurement using IVIS. Mice with SCN lesions were subjected to IVIS recoding at baseline, and then again after 3 days of treadmill exercise. (**b**) Comparison of the PER2::LUC expression rhythms of peripheral clocks in mice with SCN lesions before (SCNx-No-Ex; n = 6) or after treadmill exercise (SCNx-T-Ex; n = 6). (**c**) Plot of rhythmicity and amplitude for each mouse assessed by cosinor analysis. Blue colored symbols are before and red colored symbols are after exercise. Each symbol represents an individual mouse. (**d**) *Ex-vivo* experimental schedule. PER2::LUC bioluminescence rhythms from explants of the SCN and the liver. (**e**) Bioluminescence waveforms of the liver and SCN. (**f)** Average first peak phase of PER2:LUC rhythms indicated in (**e**). All values are represented as mean ± SEM (No-Ex, n = 4 and T-Ex, n = 4). *p < 0.05, versus No-Ex, evaluated using a *t*-test. No exercise or treadmill exercise: No-Ex or T-Ex, respectively.

Some studies have demonstrated that exercise affects the SCN-driven circadian rhythm^40^,^41^, whereas other reports did not support these findings^23^. In the current study, SCN lesions resulted in a dampened oscillation of the PER2::LUC bioluminescence rhythm, but treadmill exercise clearly restored these dampened oscillations (Fig. 6a–c and Supplementary Table S1). To examine the expression rhythms of other clock genes in various peripheral tissues, we measured mRNA of the core clock genes *Per1*, *Per2*, *Bmal1*, and *Rev-erbα* in the kidney, liver, submandibular gland, lung, and gastrocnemius muscle of mice with SCN lesions, during light (ZT5) and dark periods (ZT17). There were no significant differences in clock gene expression between ZT5 and ZT17 in No-Ex mice with SCN lesions; however, mRNA expression was upregulated or downregulated at ZT5 or ZT17 after treadmill exercise. Consequently, there were significant differences between ZT5 and ZT17 in T-Ex mice with SCN lesions (Supplementary Fig. S8a,b). The loss of day/night differences in serum corticosterone levels mice with SCN lesions was clearly restored by treadmill exercise (Supplementary Fig. S8c). In *ex-vivo* experiments, PER2::LUC rhythm in the SCN was not phase advanced by treadmill exercise, but in the liver was significantly phase advanced (Fig. 6d–f).

As it is well known that locomotor activity rhythm is controlled by SCN rhythm, we examined whether exercise during the inactive period causes a phase-advance of locomotor rhythm. Locomotor activity was acutely increased in W-Ex or T-Ex mice; however, locomotor activity in these mice returned to low levels (Supplementary Fig. S9a,b). The daily activity patterns and total activity measurements were similar before and after exercise in all (No-Ex, E-Ex, and W-Ex) groups (Supplementary Fig. S9c). These results suggest that forced treadmill exercise produced phase advances in peripheral clocks during the inactive period, independent of the SCN.

### Feeding and insulin release might not be involved in the treadmill exercise-induced phase advance of peripheral clocks

Exercise-induced changes in appetite and may increase feeding behavior after exercise. Scheduled 4 h restricted feeding for 2–7 days was shown to result in a phase advance and phase delay of the peripheral clock, when feeding time was set at daytime and nighttime, respectively^42^. Insulin release and insulin-induced phase shifts may also be involved in the restricted feeding-induced phase shift of the peripheral clock^27^,^28^,^29^,^43^. To exclude the possible effects of feeding and insulin release on the phase shift after exercise, treadmill exercise was applied at ZT4 for 1 h in mice with restricted feeding during ZT12–24 and serum insulin levels were measured. The magnitude of phase advance was comparable between the free-feeding mice and the restricted-feeding mice. In addition, insulin levels were not affected by treadmill exercise (Supplementary Fig. S10 and S11).

## Discussion

Mouse peripheral clocks are entrained by daily voluntary wheel-running and forced treadmill exercise. However, treadmill exercise, rather than wheel-running exercise, strongly entrained the peripheral clocks. Although running distance, running speed, and body temperature increases were comparable between treadmill and wheel-running exercise groups, treadmill exercise produced a strong entrainment ability, suggesting a phase shift in the peripheral clocks, which might have been because of differences in the type, rather than the intensity of exercise. The present results revealed that levels of serum corticosterone and NE in the kidney and liver were higher in the treadmill group as compared to those in the wheel-running group, which suggests that treadmill exercise may be a stressful situation, consequently resulting in big-phase shifts. Voluntary wheel-running may be a motivational and less stressful exercise compared to forced stressful exercise^30^. To clarify this hypothesis, the effect of forced and voluntary wheel-running exercise on phase shifts was examined. In the current experiment, forced wheel-running exercise produced phase advances as well as corticosterone release which was comparable to forced treadmill exercise, but not to voluntary wheel-running. Consequently, our findings emphasize the importance of the difference between forced exercise and voluntary exercise in the phase decision of peripheral clocks.

In the present experiment, we did not examine the effect of one or two days of exercise on phase-shifts. We recently reported that restraint stress-induced phase-shifts in the peripheral clocks, evaluated by IVIS, required 2–3 days for phase-shift depending on peripheral tissues^36^. Therefore, it was speculated that a similar timeframe would be required for exercise-induced entrainment. In behavioral experiments, mice can entrain their behavioral activity rhythms to both voluntary and forced scheduled locomotor exercise between 2 and 12 h each day^4^,^5^,^6^,^7^,^8^,^9^,^10^. Thus, both treadmill and wheel-running exercises play important roles in behavioral activity rhythms. In the current experiment, we examined effect of treadmill and wheel exercise on locomotor activity rhythm in the presence of a light-dark cycle. As exercise induced acute increases in locomotor activity, without affecting the daily rhythm of locomotor activity, feedback actions to the circadian clock phase may not be involved in the current experimental schedules^2^.

Voluntary free or restricted wheel-running exercise can modulate the phase of liver clock^24^,^30^,^44^. In agreement with these observations, voluntary wheel-running produced phase shifts of the peripheral clocks in the present study. However, when we compared the three different exercises in IVIS experiment, both treadmill and forced wheel-running exercise caused significantly stronger effects on phase advances than voluntary wheel-running. In addition, it has been reported that both treadmill and wheel-running exercise, for 2 h/day for 4 weeks, resulted in a similar entrainment ability of peripheral clocks in the skeletal muscle and lung^23^. In *Per2*, but not *Per1, Bmal1*, and *Rev-erbα* mRNA expression rhythms, treadmill exercise resulted in stronger phase-advances in the kidney, liver, and sub gla, compared to wheel-running. Treadmill and wheel-running produced similar phase advances in gastrocnemius muscle and lung (Supplementary Table S2). Although the reason of such organ differences (kidney, liver and sub gla vs. gastrocnemius muscle and lung) in T-Ex and W-Ex was not elucidated at present, sensitivity to exercise may be high for both exercise protocol in these exercise directly related organs. Thus, responses of clock gene expression to exercise may be dependent on the type of peripheral tissues or the specific clock genes.

We recently reported that physical as well as psychological stress could cause phase shifts in the peripheral clock of mice, depending on the timing of the stress, specifically, stress induced a phase advance during the daytime and a phase delay at nighttime^36^. These stress-induced big phase advance in peripheral clocks at ZT4–5 during inactive period is very similar to that induced by treadmill exercise. This suggests that treadmill exercise involves stress factors. Previously, we demonstrated that a core body temperature of 40–41 °C is necessary to cause a phase shift^45^. When mice were treated with exercise, their core body temperature temporarily increased to 38.5–39.0 °C (Supplementary Fig. S2), but did not reach 40 °C. Thus, the phase shift induced by exercise may not be related to increased core body temperature.

In the present study, exercise especially treadmill and forced wheel-running, increased mouse serum corticosterone and tissue NE levels, which is similar to stress application^36^. The increase in the levels of both corticosterone and NE is involved in the entrainment of peripheral clocks^33^,^36^,^37^,^46^. Circadian clock genes such as *Per1*, *Per2*, and *Npas2* are directly regulated by glucocorticoid through glucocorticoid response elements (GREs) at promoter regions^47^,^48^. Dexamethasone and stress induce a phase shift in the expression of many clock-related genes, including *Per1* and *Per2*, in several peripheral organs of mice, such as the heart, liver, and kidney^36^. Thus, exercise-induced corticosterone is important to reset the peripheral circadian rhythms. An exercise-induced increase in NE and epinephrine (E) in target tissues activates alpha- and beta-adrenoceptors, and resulted in an increase in cellular Ca^2+^ and cAMP, respectively. The elevated cAMP/Ca^2+^ levels resulted in transcriptional activation via cAMP response elements (CREs) in the *Per1* and *Per2* promoter^49^. NE injection was shown to increase *Per1* and *Per2* expression^37^ and resulted in a phase shift of *Per1* and *Per2* expression in peripheral tissues^36^,^37^. Thus, the present work is the first study elucidating the mechanistic role of corticosterone and NE in the exercise-induced entrainment of the molecular clock in peripheral organs. The expression rhythm of clock genes was advanced in the adrenal gland by treadmill or wheel exercise at ZT4–5, and the rhythm of peak level of serum corticosterone was also advanced (Supplementary Fig. S4), suggesting that exercise-induced phase advances in serum corticosterone rhythm may partially refer to the phase adjustment of the peripheral clock.

To elucidate the role of ACTH release on corticosterone levels, serum ACTH and corticosterone levels were simultaneously measured 0.5 and 1 h after exercise. ACTH release pattern was very similar in both the treadmill and wheel exercise groups; however, treadmill exercise strongly increased serum corticosterone compared to wheel exercise. Previously Lilley *et al.* reported that the rhythmic release of ACTH is not essential for rhythmic glucocorticoid secretion^50^. Light exposure is able to stimulate adrenal *Per1* expression through sympathetic activation, and the secretion of glucocorticoid, independent of ACTH^51^. These results suggest that other factors including the autonomic nervous system may be involved in corticosterone release during exercise. We examined the mRNA expression pattern of *StAR*, the key steroidogenesis rate-limiting cholesterol transporter, in the adrenal gland of exercise-treated mice, because *StAR* is rhythmically expressed under the direct regulation of adrenal clock proteins^38^,^39^. Although the expression rhythm of clock genes was clearly advanced by exercise, the *StAr* gene expression rhythm was unaffected by three days of treadmill or wheel exercise, suggesting that more time might be required to observe changes in *StAr* gene expression. Alternatively, this gene might only minimally respond to exercise.

To confirm that the current exercise protocol was sufficient to cause a phase-shift of the peripheral clock, mice were subjected to three consecutive days of treadmill exercise and the maintenance of the phase shift was examined after suspending exercise for one or three days. The phase advance was maintained on Day 1 of suspension, but advance disappeared on Day 3 of suspension. This result strongly suggests that present exercise protocols produce enough stimuli for entrainment of peripheral clock oscillation. Corticosterone levels were high at ZT5 after only 1 h of exercise on Day 3, but they were low at ZT5 on Day1 and 3 with exercise suspension, suggesting that acute corticosterone release after exercise may be important to cause phase-shifts.

The present findings demonstrated that treadmill exercise-induced phase advance was blocked by ADX in the sub gla clock, and completely blocked by ADX and NE receptor blockers in the kidney and liver clocks (Fig. 5). Thus, blockade of corticosterone pathway is not sufficient to block the exercise-induced phase advances of kidney and liver clocks; however, blockade of corticosterone is enough to block that of the sub gla clock. The basal levels of NE were higher in the sub gla than in kidney and liver, and the levels of NE after treadmill exercise were not significantly increased in the sub gla (Fig. 5). Thus, the NE response system to treadmill exercise may be weak in the sub gla. In all peripheral clocks, exercise-induced phase advances were completely blocked by ADX and NE receptor blockers. Actually, NE release was still observed in ADX mice after exercise, and corticosterone release was also observed in mice treated with an NE receptor blocker (Fig. 6). Thus, both adrenal and sympathetic nervous systems may be cooperatively involved in the exercise-induced phase shift. Wheel-running exercises for 10 days decreased the magnitude of the phase shift, which was accompanied by a decrease in corticosterone and NE levels. This exercise tolerance is similar to stress tolerance^36^,^52^.

Scheduled 4 h restricted feeding for 2–7 days could phase advance and phase delay the peripheral clocks, when feeding time was set at daytime and nighttime, respectively^42^. Scheduled feeding, physical stress, and treadmill exercise did not affect the phase of the SCN clock^27^,^36^ (Fig. 3 in the present result). Thus, the phenomenon of phase shifts induced by restricted feeding was very similar to that induced by treadmill exercise. This suggests that similar and common signaling pathways might be involved in the entrainment of the peripheral clocks in mice undergoing restricted feeding and exercise. Insulin release and the insulin-induced phase shift may be involved in the restricted feeding-induced phase shift of the peripheral clocks^27^,^28^,^29^,^43^. In the current study, treadmill exercise under a 12 h restricted feeding schedule, during the nighttime, produces a phase advance similar to that observed in free-feeding conditions. In addition, treadmill exercise did not increase serum insulin levels. Taken together, our results strongly suggest that feeding and/or insulin release may not be involved in the exercise-induced phase shifts.

Exercise, as a non-photic stimulus, affects *Per1* and *Per2* gene expression, and ERK phosphorylation in the SCN^2^. The PER2::LUC peak time in the SCN was not significantly different between mice subjected to various wheel-access conditions (free access, scheduled 6 h access at early night, or scheduled 6 h access at late night)^24^. In contrast, in the liver, early night and late night wheel-running delayed the timing of the peaks^24^. Using the same PER2::LUC mice, Pendergast *et al.* recently reported that free access of wheel-running delayed the liver clock, without affecting the phase of the SCN clock^30^. These studies provide evidence that free access or nighttime access to wheel-running affects the phase of peripheral clocks without affecting the phase of the SCN clock. Our current treadmill exercise schedule during the daytime also caused a phase advance of the liver clock without affecting the SCN clock in *ex-vivo* experiments. An SCN lesion flattened the PER2::LUC rhythm and the mRNA expression rhythms of clock genes in various peripheral tissues including the lung and skeletal muscle. Exercise resulted in reappearance of the PER2::LUC rhythm as well as *Per1, Per2, Bmal1*, and *Rev-erbα* mRNA rhythms and also the corticosterone rhythm in mice with SCN lesions (Fig. 3, Supplemental Fig. S5). These data support the notion that exercise induces the entrainment of the peripheral clocks independent of the SCN-driven circadian rhythm. A previous study also found that treadmill or wheel-running during the daytime did not affect the phase of the SCN clock^23^. Based on these reports and our present results, it is likely that the SCN is sufficient, but is not necessary, for the entrainment of peripheral clocks by exercise.

Exercise can entrain the skeletal muscle and lung clocks, organs directly impacted by exercise^23^,^25^. In the present experiment, exercise was conducted only for 3 days, therefore phase advance effect may be weak in muscle clock with no differences between T-Ex and W-Ex. In general, the liver is sensitive to the restricted feeding-induced phase shift^35^,^36^. In the current experiments, responses of mRNA expression to exercise were observed in all peripheral clocks; however, the intensity of responses was dependent on the specific clock gene and the peripheral tissue. Among peripheral clocks, the kidney was more sensitive to exercise than the liver and the Sub gla in the IVIS experiment. The kidney clock is also sensitive to stress^24^. Thus, the kidney may be sensitive to forced exercise in a manner similar to stress signals.

In human experiments, exercise is a potent stimulus for cortisol secretion and elevates circulating NE and epinephrine concentrations^53^,^54^. In addition, the re-entrainment of the sleep-wake cycle to a phase advanced schedule is accelerated by timed physical exercise^55^. The physiological responses of the neuroendocrine system in response to acute exercise are dependent on the intensity and the volume (sets x repetitions x intensity) of the exercise performed in humans^53^. However, there are no reports comparing the effects of voluntary and forced exercise on the neuroendocrine system in humans. Future studies will be directed towards examining whether voluntary and forced exercise differentially affect the circadian rhythm in humans.

In summary, the present results suggest that forced exercise, rather than voluntary exercise, produces phase shifts depending on the time of exercise. The release of both corticosterone and NE may be involved in forced exercise-induced phase shifts of peripheral clocks.

## Methods

### Animals

Heterozygous PER2::LUC knock-in mice^56^ in the ICR background were used in this study. Mice were kept in a room maintained on a 12 h light/12 h dark (LD) cycle (lights on from 08:00 to 20:00). Zeitgeber time 0 (ZT0) was designated as lights-on time and ZT12 as lights-off time during the LD cycle. The mice were housed individually in plastic cages, at a temperature of 22 ± 2 °C, humidity of 60 ± 5%, and a light intensity of 100–150 lux at cage level. The mice were provided with a standard diet (MF; oriental Yeast Co. Ltd, Tokyo, Japan) and water *ad libitum*. The procedures conformed to the “Fundamental Guidelines for Proper Conduct of Animal Experiment and Related Activities in Academic Research Institutions” (published by the Ministry of Education, Culture, Sports, Science and Technology, Japan) and were approved by the Committee for Animal Experimentation of the School of Science and Engineering at Waseda University (permission #2015-A044).

### Forced Exercise

Mice ran on the treadmill (Exer-6 M Treadmill, Columbus Instrument, OH, USA) for 1 h at a speed of 9–15 m/min. This treadmill speed was chosen on the basis of a previous report^57^ with a goal of trying to match running intensities for the first 3 days in our experimental with those previously reported for voluntary wheel-running. Mice were adapted to the treadmill apparatus for 30 to 60 min on the day before the 4 to 7 days of test experiments. Mice were removed from their home cages and placed on the treadmill from ZT4 to 5 or ZT20 to 21. No-Ex mice for the non-exercise control were taken out of their home cage for 1 h. In a recent paper, we demonstrated that wake-up for several hours during the light phase did not affect the phase of peripheral clocks, evaluated by IVIS^36^.

### Voluntary wheel-running exercise

Each mouse was housed in a cage with a wheel that could be locked or unlocked using a timer. The wheel was composed of a stainless steel wire (diameter, 11.5 cm; STARR Life Sciences Corp., Oakmont, PA). Mice had free access to the wheel from ZT4 to 5, or ZT20 to 21, and the wheel was locked at other times.

### Forced wheel-running exercise

For forced wheel-running, we used the Mouse Forced Exercise Walking Wheel System, diameter, 15.1 cm (Lafayette Instruments, Lafayette, USA). Running conditions for both treadmill and voluntary wheel-running were 12 m/min for 1 h. However, under the same speed, mice tended to drop off the wheel ring and escaped from running. To avoid such behavior, the mice were kept in the forced wheel for 1.5 h, at a speed of 8 m/min so that mice could run along with the wheel rotations. Mice were removed from home cages and placed on the forced wheel from ZT 4 to 5.5.

### *In vivo* monitoring of PER2::LUC bioluminescence

*In vivo* monitoring of PER2::LUC bioluminescence was performed as previously described^26^ using an IVIS kinetics system (Caliper Life Sciences, Hopkinton, MA, USA, and Summit Pharmaceuticals International Corporation, Tokyo, Japan). Mice were anesthetized with isoflurane (Mylan Inc., Tokyo, Japan) and concentrated oxygen in an induction chamber. Anesthetized mice were injected subcutaneously on the back near the neck with D-luciferin potassium salt (15 mg/kg; Promega, WI, USA). Bioluminescence images with 1 min exposure were taken for the dorsal-up position at 6 min or 8 min after luciferin injection for the kidney, and at 10 min or 12 min after luciferin injection in the ventral-up position for the liver and the submandibular gland. Images were obtained six times a day at 4 h intervals (ZT9, 13, 17, 21, 1, and 5). Mice were returned to their home cages during each interval of the imaging sessions. Photon counts from each tissue were analyzed using the Living Image 3.2 software (Perkin Elmer). The average photon/s value of the data from the 6 points for each day was designated as 100%, and the bioluminescence rhythm was expressed as a percentage of each set of 6 points for individual organs. The peak phase, amplitude, and rhythmicity of normalized data were determined using the single cosinor procedure program (Acro.exe, version 3.5; See ref. 58).

### SCN Lesion

A bilateral thermal lesion of the SCN was made after midazolam/xylazine-induced anesthesia, as described previously^26^. A stainless steel electrode (0.35 mm in diameter) was inserted bilaterally into the SCN (0.5 mm anterior and ± 0.2 mm lateral to the bregma and 5.8 mm from the skull surface) with a thermal lesioning device (RFG-4 A; Muromachi). A lesion was made by maintaining a temperature of 55 °C for 7 s using a current path. Mice with SCN lesions were used for experiments after a recovery period of at least one week.

### *Ex vivo* recording of bioluminescence rhythm from tissue slices

An *ex vivo* luciferase assay with tissue slices was performed as previously described^26^. The SCNs from PER2::LUC mice were sliced to a thickness of 300 μm with a vibratome (DTK-1500, D.S.K, Kyoto, Japan). SCN slices on a Millicell-CM culture insert (Merck Millipore, MA, USA), and small liver slices were explanted into a 35 mm petri dish (AGC Techno Glass Co., LTD., Tokyo, Japan). These tissues were cultured in 1.3 mL Dulbecco modified Eagle medium (Invitrogen) and D-luciferin potassium (Promega, WI, USA), and incubated at 37 °C. Bioluminescence was monitored for 1 min at 10 min intervals with a dish-type luminometer (LumiCycle, Actimetrics, IL, USA).

### Enzyme-linked immunosorbent assay

Levels of specific antibodies, corticosterone, ACTH, and insulin were measured in serum samples by an enzyme-linked immunosorbent assay (ELISA). We used a corticosterone ELISA kit (ASSAYPRO, St. Charles, MO), ACTH ELISA kit (Phoenix Pharmaceuticals, Burlingame, California), and an insulin ELISA kit (Mercodia, Uppsala, Sweden). Assays were performed according to the manufacturers’ instructions.

### High performance liquid chromatography-electrochemical detection (HPLC-ECD)

Tissue monoamine content was measured by HPLC-ECD (HTEC 500) (Eicom, Kyoto, Japan). At ZT5 or 21, No-Ex, W-Ex, and T-Ex mice were anesthetized with isoflurane and their livers, kidneys and submandibular glands were removed. To each tissue sample, 0.2 M perchloric acid (including 100 μM EDTA·2Na) and 20 ng of isoproterenol were added. Samples were homogenized using a micro-homogenizer and then centrifuged at 15,000 *g* at 4 °C for 15 min. The supernatants collected for each sample were filtered using a 0.45 μm filter. The quantity of monoamine in each 20 μl sample was measured using HPLC-ECD with the following conditions: the transfer phase consisted of 85% 0.1 M acetate citric acid buffer (pH 3.5) containing 5 mg/L EDTA·2Na, 190 mg/L 1-octanesulfonic acid sodium salt, and 15% methanol (99% purity); the velocity of the flow was 500 μL/min; the column temperature was set to 25 °C; the applied voltage was set to +750 mV versus Ag/AgCl. The data were analyzed with EPC-300 software (Eicom). Epinephrine could not be detected in the submandibular gland (Sub gla).

### Drug treatment

Prazosin hydrochloride (1 mg/kg) (Wako Pure Chemical Industries Ltd, Osaka, Japan), and propranolol hydrochloride (2 mg/kg) (TOKYO KASEI, Tokyo, Japan) were used in this experiment. Saline was used for the vehicle treatment.

### Adrenalectomy

Adrenalectomy was carried out by the dorsal approach under midazolam/xylazine-induced anesthesia. The skin on the back was shaved and disinfected, and an incision of approximately 1 cm was made above and parallel to the spinal cord. The adrenal glands were removed from the surrounding fat tissue via small openings made in the muscle layer to the left and right of the spinal cord. ADX mice were provided with 0.9% NaCl water *ad libitum* to maintain the appropriate mineral balance. ADX mice were used for experiments after a recovery period of at least one week.

### Measurement of the core body temperature

A small temperature sensor (Thermochron SL; KN Laboratories Inc., Osaka, Japan), shaped similar to a button battery, was used to record the core body temperature. One side of the sensor was covered with a plastic shield and the other side served as the sensing surface. The mice were anesthetized using midazolam/xylazine and the sensor was implanted. The sensing surface was placed near the liver and the covered surface was located just under the skin. This specific positioning prevented the sensor from detecting changes in the environmental temperature. Mice were used for the experiment after a recovery period of at least one week. Mice were recorded automatically at 10 min intervals for 2 weeks. The recordings were analyzed using RhManager (Version 2.10; N Laboratories Inc., Osaka, Japan).

### Real-time RT-PCR

RNA was extracted from peripheral tissues using phenol (Omega Bio-Tek Inc., Norcross, GA, USA). Real-time reverse transcription PCR was performed using the One-Step SYBR RT-PCR Kit (Takara Bio Inc., Shiga, Japan) with specific primer pairs (listed in Supplementary Table S3) on a Piko Real PCR system (Thermo Fisher Scientific, Waltham, MA, USA). Primers were designed using Primer 3 software^59^,^60^. The relative expression levels of target genes were normalized to that of *Gapdh*. Data were analyzed using the ΔΔCt method. A melt curve analysis of each primer was performed to identify non-specific products. Details of methods were published in our previous papers^36^.

### Activity Monitoring

General locomotor activity was monitored using an area sensor (F5B, Omron, Tokyo, Japan) and analyzed using ClockLab software (Actimetrics, Wilmette, IL) as previously described^26^. Locomotor activity was monitored before and during three consecutive days of forced treadmill exercise or wheel-running exercise.

### Statistical analysis

Data are expressed as mean ± SEM values. Statistical analysis was performed using the GraphPad Prism version 6.03 (GraphPad Software, San Diego, CA). We determined if data showed normal or non-normal distribution, and equal or biased variation using the D’Agostino-Pearson test/Kolmogorov-Smirnov test and the F value test/Bartlett’s test, respectively. Parametric analysis was conducted using a one-way or two-way ANOVA with a Tukey test, Sidak test, or Student’s *t*-test for *post-hoc* analysis. Non-parametric analysis was done using the Kruskal-Wallis test with the Dunn’s test or the Mann-Whitney test for *post-hoc* analysis. Data from rhythmicity experiments were analyzed using the Fisher’s exact test.

## Additional Information

**How to cite this article**: Sasaki, H. *et al.* Forced rather than voluntary exercise entrains peripheral clocks via a corticosterone/noradrenaline increase in PER2::LUC mice. *Sci. Rep.* **6**, 27607; doi: 10.1038/srep27607 (2016).

## Supplementary Material

Supplementary Information
