# Supplementary material for: Forced rather than voluntary exercise entrains peripheral clocks via a corticosterone/noradrenaline increase in PER2::LUC mice
Source: Sci Rep. 2016 Jun 8;6:27607. doi: 10.1038/srep27607 (PMC4897787; doi:10.1038/srep27607)
Supplement: Supplementary Information [file srep27607-s1.doc]

Supplementary Information for

Forced rather than voluntary exercise entrains peripheral clocks via a corticosterone/noradrenaline increase in PER2::LUC mice

Hiroyuki Sasaki, Yuta Hattori, Yuko Ikeda, Mayo Kamagata, Shiho Iwami, Shinnosuke Yasuda, Yu Tahara and Shigenobu Shibata

This PDF file includes

Figs. S1 to S7

Table. S1,S2, S3

**
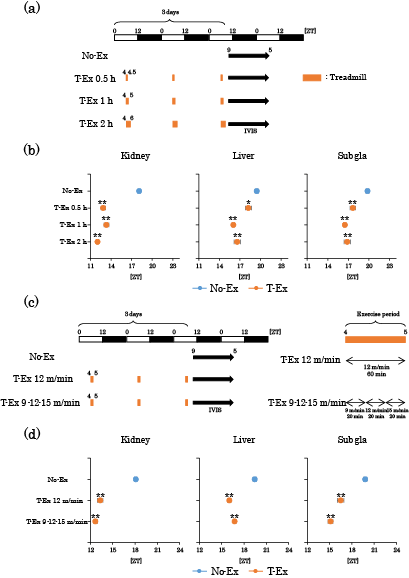
**

**Supplementary Figure S1 Effect of treadmill exercise duration time and speed of exercise on phase-advance of peripheral clocks**

(**a**) Experimental schedule. The white and black bars indicate environmental 12 h light and dark conditions. The orange short bar indicates exercise periods. The black arrow indicates the period of measurement using the *in vivo* imaging system (IVIS). (**b**) Average peak phase of PER2::LUC rhythms in each tissue after treadmill exercise (T-Ex) for 0.5, 1, or 2 h for 3 consecutive days. Data from (**b**) are represented as mean ± SEM (No-Ex, n=8; T-Ex 0.5 h, n=6; T-Ex 1 h, n=6; T-Ex 2 h, n=5). (**c**) Experimental schedule. The white and black bars indicate environmental 12 h light and dark conditions. The orange short bar indicates exercise periods. The black arrow indicates the period of measurement using IVIS. (**d**) Average peak phase of PER2::LUC rhythms in each tissue after treadmill exercise (T-Ex) for 1 h with a change of speed for 3 consecutive days. Data (**d**) are represented as mean ± SEM (No-Ex, n=8; T-Ex 12 m/min, n=6; T-Ex 9-12-15 m/min, n=6). ** p<0.01, * p<0.05 versus No-Ex, evaluated using the one-way ANOVA test with Tukey *post-hoc* test. No exercise, treadmill exercise, or wheel-running exercise: No-Ex, T-Ex, or, W-Ex, respectively.

**
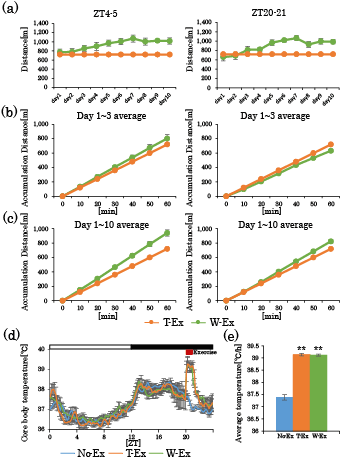
**

**Supplementary Figure S2 Running distance, speed, and body temperature increase in treadmill or wheel exercise**

(**a**) Daily running distance (**b**) Accumulated running distance for 60 min. Data are averaged values of 3 consecutive days of exercise. (**c**) Accumulated running distance for 60 min. Data are averaged values of 10 days of exercise. (**d**) Daily body temperature rhythm in No-Ex, T-Ex, or W-Ex groups. (**e**) Average body temperature during exercise for ZT20-21. All values are represented as mean ± SEM (No-Ex, n=6; W-Ex, n=4; T-Ex, n=6). ** p<0.01 versus No-Ex, evaluated using the one-way ANOVA test with Tukey *post-hoc* test. No exercise, treadmill, or wheel exercise: No-Ex, T-Ex, or W-Ex, respectively.

**
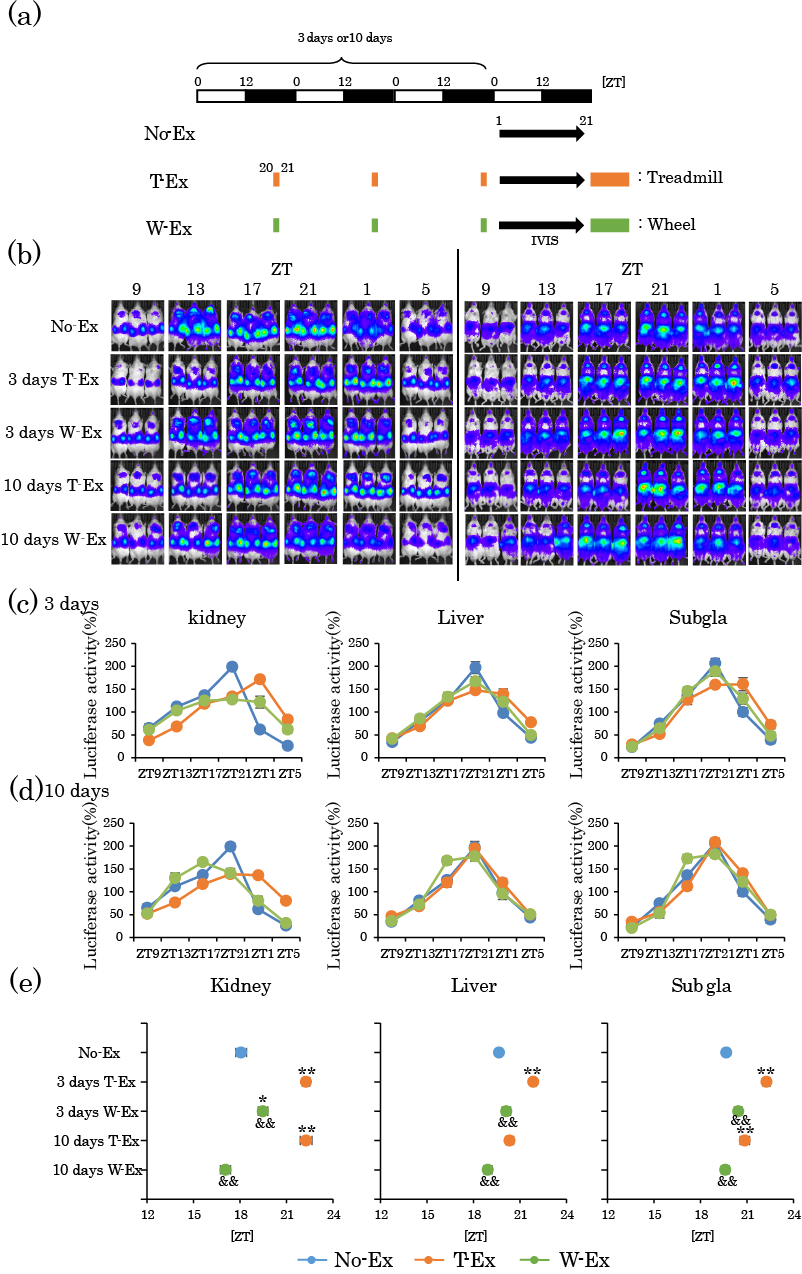
**

**Supplementary Figure S3 Treadmill or wheel-running exercise during the activity period produces a phase delay of mouse peripheral clocks**

(**a**) Experimental schedule. The white and black bars indicate environmental 12 h light and dark conditions. The orange short bar indicates forced exercise periods. The green short bar indicates voluntary exercise period. The black arrow indicates the period of measurement using IVIS. (**b**) Representative images of *in vivo* PER2::LUC bioluminescence in the kidney (left panels) and in liver and submandibular gland (right panels). (**c**) and (**d**) Comparison of PER2::LUC expression rhythms of peripheral clocks in mice after non-exercise (No-Ex), treadmill exercise (T-Ex), or wheel-running exercise (W-Ex). Exercise was applied for 3 days (**c**) or 10 days (**d**). (**e**) Average peak phase of PER2::LUC rhythms in each tissue after exercise indicated in (**c**) and (**d**). All values are represented as mean ± SEM (No-Ex, n=8; 3 days T-Ex, n=6; 3 days W-Ex, n=5; 10 days T-Ex, n=6; 10 days W-Ex, n=5). ** p<0.01 * p<0.05 versus No-Ex, evaluated using the one-way ANOVA test with Tukey *post-hoc* test. && p<0.01 & p<0.05 versus T-Ex at same period and span, evaluated using the one-way ANOVA test with Tukey *post-hoc* test. No exercise, treadmill exercise, or wheel-running exercise: No-Ex, T-Ex, or W-Ex, respectively.


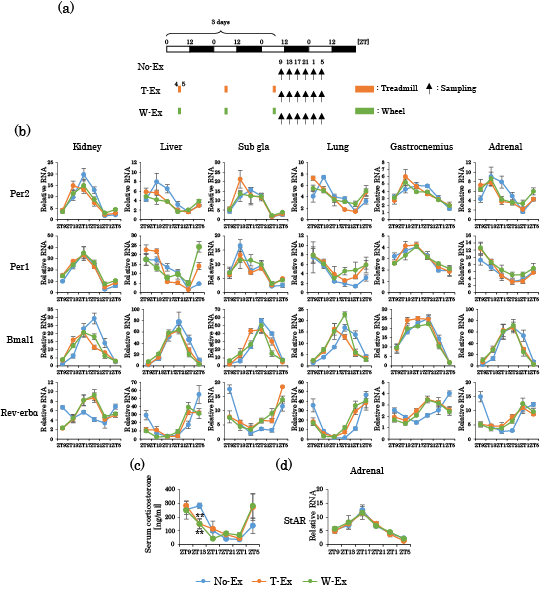


**Supplementary Figure S4 Treadmill or wheel-running exercise during the inactive period for 3 consecutive days causes phase-advance in various peripheral tissues and clock genes.**

(**a**) Experimental schedule. The white and black bars indicate environmental 12 h light and dark conditions. The orange short bar indicates forced exercise periods. The green short bar indicates voluntary exercise period. The black arrow indicates the sampling time. (**b**) RNA expression profiles of clock genes in the kidney, liver, submandibular gland, lung, gastrocnemius, and adrenal gland. (**c**) Time course of serum corticosterone levels after 3 consecutive days of treadmill or wheel running exercise. (**d**) mRNA expression profiles of corticosterone synthesizing gene, *StAR*, in adrenal gland. All values are represented as mean ± SEM (n=4 at each time point; total n=4 × 6 points for No-Ex, T-Ex, and W-Ex groups). ** p<0.01 versus No-Ex, evaluated using the one-way ANOVA test with Tukey *post-hoc* test. No exercise, treadmill exercise, or wheel-running exercise: No-Ex, T-Ex, or W-Ex, respectively.

**
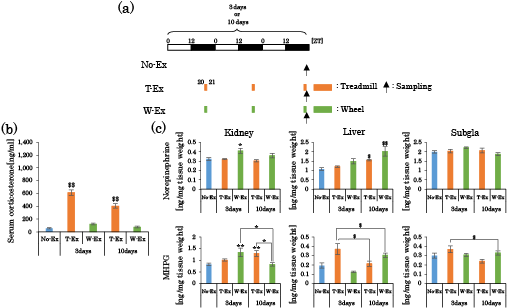
**

**Supplementary Figure S5 Treadmill or wheel-running exercise during the active period increases the levels of serum corticosterone and peripheral tissue NE/MHPG**

(**a**) Experimental schedule. The white and black bars indicate environmental 12 h light and dark conditions. The orange short bar indicates forced exercise periods. The green short bar indicates voluntary exercise periods. The black arrow indicates the sampling time. (**b**) Serum corticosterone levels after 3 days or 10 days of treadmill exercise or wheel-running exercise. (**c**) NE and MHPG levels in the kidney, liver, and Sub gla after 3 days or 10 days of treadmill exercise or wheel-running exercise. All values are represented as mean ± SEM (No-Ex, n=13; 3 days T-Ex, n=5; 3 days W-Ex, n=5; 10 days T-Ex, n=6; 10 days W-Ex, n=6). ** p<0.01 * p<0.05 versus No-Ex, evaluated using the one-way ANOVA test with Tukey *post-hoc* test. $$ p<0.01 $ p<0.05 versus No-Ex, evaluated using the Kruskal-Wallis test with Dunn *post-hoc* test. No exercise, treadmill exercise, or wheel-running exercise: No-Ex, T-Ex, or W-Ex, respectively.

**
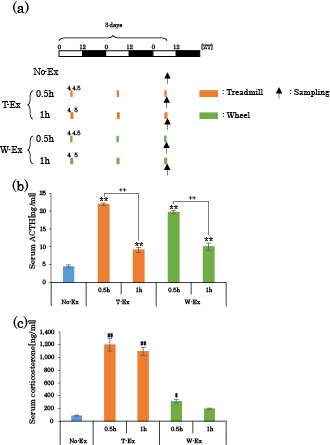
**

**Supplementary Figure S6 Treadmill or wheel-running exercise for 0.5 and 1 h during the inactive period increases the levels of serum ACTH and corticosterone.**

(**a**) Experimental schedule. The white and black bars indicate environmental 12 h light and dark conditions. The orange short bar indicates forced exercise periods. The black arrow indicates the sampling time. (**b**) Serum ACTH levels 0.5 and 1 h after 3 consecutive days of treadmill exercise or wheel running exercise. (**b**) Serum corticosterone levels 0.5 and 1 h after 3 consecutive days of treadmill exercise or wheel running exercise. All values are represented as mean ± SEM (No-Ex, n=13; 0.5 h T-Ex, n=6; 1 h T-Ex, n=6; 0.5 h W-Ex, n=6; 1 h W-Ex, n=6). ** p<0.01 versus No-Ex, ++ p<0.01, evaluated using the one-way ANOVA test with Tukey *post-hoc* test. $$ p<0.01 $ p<0.05, evaluated using the Kruskal-Wallis test with Dunn *post-hoc* test. No exercise, treadmill exercise, or wheel-running exercise: No-Ex, T-Ex, or W-Ex, respectively.


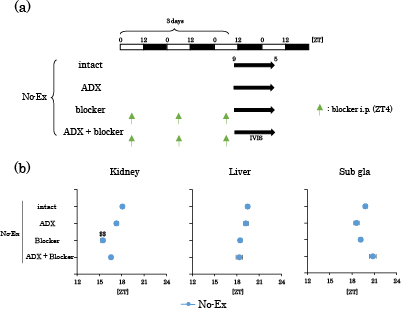


**Supplementary Figure S7 Effect of adrenalectomy and/or adrenergic receptor blockers on the phase of peripheral clocks under non-exercise conditions**

(**a**) Experimental schedule. The white and black bars indicate environmental 12 h light and dark conditions. The black arrow indicates the period of measurement using IVIS. The green upward arrow indicates injection time of the blocker. ADX, adrenalectomized mice. Blocker, prazosin hydrochloride (1 mg/kg i.p.) and propranolol hydrochloride (2 mg/kg i.p.) were administered at ZT4 for 3 consecutive days. (**b**) Average peak phase of PER2::LUC rhythms in each tissue under different experimental conditions. All values are represented as mean ± SEM (No-Ex intact, n=8; No-Ex ADX, n=4; No-Ex Blocker, n=6; No-Ex ADX + Blocker, n=6). $$ p<0.01 versus No-Ex, evaluated using the Kruskal-Wallis test with Dunn *post-hoc* test.


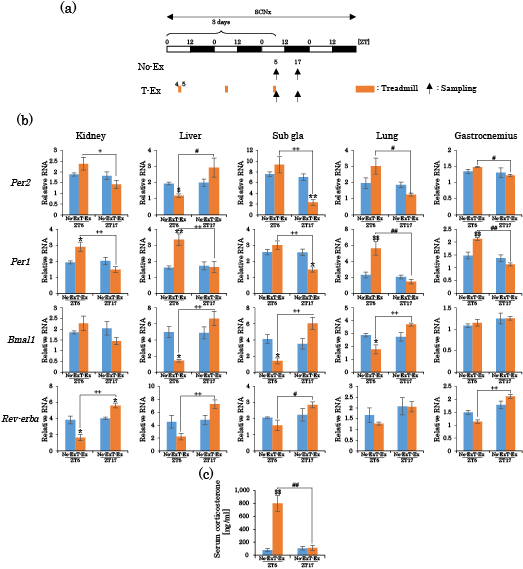


**Supplementary Figure S8 Treadmill exercise during the inactive period reproduces rhythmicity of clock gene expression in mice with SCN lesions.**

(**a**) Experimental schedule. The white and black bars indicate environmental 12 h light and dark conditions. The orange short bar indicates forced exercise periods. The black arrow indicates the sampling time. (**b**) Day/night mRNA expression profiles of clock genes of the kidney, liver, submandibular gland, lung, and gastrocnemius muscle in mice with SCN lesions after 3-day treadmill exercise. (**c**) Serum corticosterone levels after 3-day exercise in mice with SCN lesions. All values are represented as mean ± SEM (n=4 at each time point; total n=4 × 2 points for No-Ex and T-Ex groups). ** p<0.01 * p<0.05 versus No-Ex at the same time (ZT5 or ZT17), ++ p<0.01 + p<0.05 versus ZT5 at the same exercise condition (No-Ex or T-Ex), evaluated using the two-way ANOVA test with Tukey *post-hoc* test. $$ p<0.01 $ p<0.05 versus No-Ex at the same time (ZT5 or ZT17), ## p<0.01 # p<0.05 versus ZT5 at the same exercise condition (No-Ex or T-Ex), evaluated using the Mann-Whitney test. No exercise or treadmill exercise: No-Ex or T-Ex, respectively.


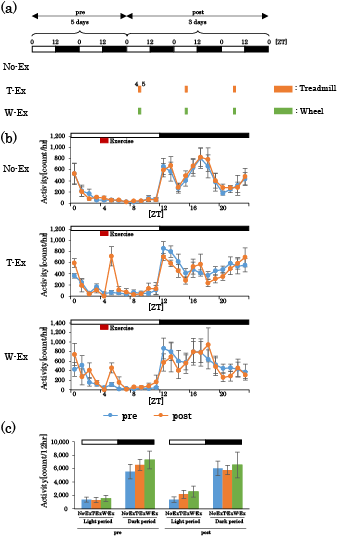


**Supplementary Figure S9 Treadmill or wheel-running exercise during the inactive period for 3 consecutive days does not affect locomotor activity rhythm.**

(**a**) Experimental schedule. The white and black bars indicate environmental 12 h light and dark conditions. The orange short bar indicates forced exercise periods. The green short bar indicates voluntary exercise periods. (**b**) Daily pattern of locomotor activity in the No-Ex, T-Ex, and W-Ex groups before exercise (pre, 5 days) or during exercise (post, 3 days). (**c**) Average locomotor activity during the light period (ZT0-12) and dark period (ZT12-0), before exercise (pre) or during exercise (post). All values are represented as mean ± SEM (No-Ex, n=7; T-Ex, n=5; W-Ex, n=5).


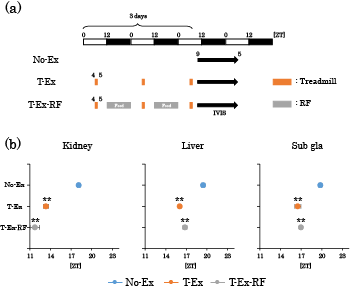


**Supplementary Figure S10 Treadmill exercise during the inactive period produces phase advance of mouse peripheral clocks under 12 h restricted feeding during the active period**

(**a**) Experimental schedule. The white and black bars indicate environmental 12 h light and dark conditions. The orange short bar indicates exercise periods. Horizontal gray bars indicate restricted feeding periods. The black arrow indicates the period of measurement using IVIS. RF: restricted feeding during activity period. (**b**) Average peak phase of PER2::LUC rhythms in each tissue after treadmill exercise during 12 h restricted feeding. All values are represented as mean ± SEM (No-Ex, n=8; T-Ex, n=6, T-Ex-RF, n=5). ** p<0.01 versus No-Ex, evaluated using the one-way ANOVA test with Tukey *post-hoc* test. No exercise or treadmill exercise: No-Ex or T-Ex respectively.


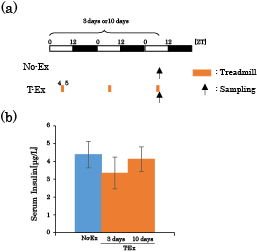


**Supplementary Figure S11 Treadmill exercise during the inactive period did not increase the levels of serum insulin**

(**a**) Experimental schedule. The white and black bars indicate environmental 12 h light and dark conditions. The orange short bar indicates exercise periods. The black upward arrow indicates the sampling time. (**b**) Serum insulin levels after treadmill exercise for 3 or 10 consecutive days. All values are represented as mean ± SEM (No-Ex, n=8; 3 days T-Ex, n=4; 10 days T-Ex, n=6). No exercise or treadmill exercise: No-Ex or T-Ex respectively.

**Supplementary Table S1. Summary of cosinor analysis of IVIS data in each experiment.**

|  | group | kidney | | liver | | sub gla | |
| --- | --- | --- | --- | --- | --- | --- | --- |
|  |  | rhythmic/total^(a)^ | amplitude | rhythmic/total | amplitude | rhythmic/total | amplitude |
| Fig. 1 | No-Ex | 8/8 | 66.1 ± 6.4 | 8/8 | 78.4 ± 7.5 | 8/8 | 89.7 ± 7.0 |
|  | 3 days T-Ex | 6/6 | 61.1 ± 6.9 | 6/6 | 64.5 ± 6.2 | 6/6 | 68.9 ± 6.8 |
|  | 3 days W-Ex | 6/6 | 74.4 ± 3.3 | 6/6 | 72.8 ± 3.7 | 6/6 | 79.1 ± 4.5 |
|  | 10 days T-Ex | 6/6 | 64.6 ± 3.7 | 6/6 | 53.7 ± 2.2^##^ | 6/6 | 59.2 ± 4.5^##^ |
|  | 10 days W-Ex | 5/5 | 69.7 ± 4.4 | 5/5 | 72.8 ± 4.3 | 5/5 | 84.2 ± 5.8 |
| Fig. 2 | No-Ex^(b)^ | 8/8 | 66.1 ± 6.4 | 8/8 | 78.4 ± 7.5 | 8/8 | 89.7 ± 7.0 |
|  | T-Ex^(b)^ | 6/6 | 61.1 ± 6.9 | 6/6 | 64.5 ± 6.2 | 6/6 | 68.9 ± 6.8 |
|  | vW-Ex^(b)^ | 6/6 | 74.4 ± 3.3 | 6/6 | 72.8 ± 3.7 | 6/6 | 79.1 ± 4.5 |
|  | fW-Ex | 6/6 | 67.0 ± 5.6 | 6/6 | 61.7 ± 3.3^#^ | 6/6 | 66.0 ± 3.9 |
| Fig. 4 | No-Ex intact | 8/8 | 77.1 ± 7.5 | 8/8 | 87.4 ± 4.3 | 8/8 | 94.0 ± 4.3 |
|  | T-Ex intact | 6/6 | 62.6 ± 6.5 | 6/6 | 65.7 ± 6.7 | 6/6 | 74.2 ± 7.2 |
|  | T-Ex ADX | 6/6 | 54.9 ± 3.9 | 6/6 | 58.5 ± 6.0 | 6/6 | 69.3 ± 3.5 |
|  | T-Ex Blocker | 6/6 | 51.4 ± 3.1 | 6/6 | 62.6 ± 3.3 | 6/6 | 71.7 ± 4.1 |
|  | T-Ex ADX+Blocker | 5/6 | 52.6 ± 5.1 | 5/6 | 68.9 ± 8.0 | 5/6 | 63.6 ± 4.2^*^ |
| Fig. 6 | Intact-No-Ex^(b)^ | 8/8 | 66.1 ± 6.4 | 8/8 | 78.4 ± 7.5 | 8/8 | 89.7 ± 7.0 |
|  | SCNx-No-Ex | 1/6^++^ | 31.0 ± 3.0^**^ | 1/6^++^ | 28.7 ± 1.1^##^ | 0/6^++^ | 34.0 ± 4.7^**^ |
|  | SCNx-T-Ex | 5/6^&^ | 46.6 ± 3.3^**,$$^ | 6/6^&^ | 55.4 ± 4.4 | 6/6^&&^ | 61.1 ± 5.1^**,$$^ |
| Fig. S1 | No-Ex^(b)^ | 8/8 | 66.1 ± 6.4 | 8/8 | 78.4 ± 7.5 | 8/8 | 89.7 ± 7.0 |
|  | 0.5 h T-Ex | 6/6 | 63.5 ± 2.9 | 6/6 | 63.5 ± 3.3 | 6/6 | 72.1 ± 6.0 |
|  | 1 h T-Ex^(b)^ | 6/6 | 61.1 ± 6.9 | 6/6 | 64.5 ± 6.2 | 6/6 | 68.9 ± 6.8 |
|  | 2 h T-Ex | 5/5 | 55.7 ± 2.3^**^ | 5/5 | 50.4 ± 1.8^##^ | 5/5 | 55.1 ± 3.4^##^ |
|  | 9-12-15 m/min T-Ex | 6/6 | 58.5 ± 2.1 | 6/6 | 59.8 ± 3.8 | 6/6 | 74.7 ± 6.3 |
| Fig. S3 | No-Ex | 8/8 | 65.8 ± 3.7 | 8/8 | 69.6 ± 8.8 | 8/8 | 83.6 ± 10.3 |
|  | 3 days T-Ex | 6/6 | 66.6 ± 2.2 | 6/6 | 56.4 ± 3.7^*^ | 6/6 | 74.0 ± 6.1 |
|  | 3 days W-Ex | 5/5 | 52.2 ± 4.0^*^ | 5/5 | 68.8 ± 2.8 | 5/5 | 91.5 ± 3.9 |
|  | 10 days T-Ex | 6/6 | 46.0 ± 2.1^**^ | 6/6 | 76.7 ± 2.9 | 6/6 | 87.8 ± 3.8 |
|  | 10 days W-Ex | 6/6 | 68.5 ± 2.8 | 6/6 | 79.5 ± 3.5 | 6/6 | 84.5 ± 3.4 |
| Fig. S7 | No-Ex intact^(c)^ | 8/8 | 77.1 ± 7.5 | 8/8 | 87.4 ± 11.4 | 8/8 | 94.0 ± 4.3 |
|  | No-Ex ADX | 4/4 | 71.3 ± 5.3 | 4/4 | 74.4 ± 5.6 | 4/4 | 95.2 ± 7.6 |
|  | No-Ex Blocker | 6/6 | 52.7 ± 5.9 | 6/6 | 64.0 ± 4.7 | 6/6 | 67.9 ± 5.6 |
|  | No-Ex ADX + Blocker | 6/6 | 62.9 ± 5.2 | 6/6 | 57.0 ± 4.3 | 6/6 | 60.8 ± 6.3^*^ |
| Fig. S10 | No-Ex^(b)^ | 8/8 | 66.1 ± 6.4 | 8/8 | 78.4 ± 7.5 | 8/8 | 89.7 ± 7.0 |
|  | T-Ex^(b)^ | 6/6 | 61.1 ± 6.9 | 6/6 | 64.5 ± 6.2 | 6/6 | 68.9 ± 6.8 |
|  | T-Ex-RF | 5/5 | 54.6 ± 3.4^**^ | 5/5 | 55.0 ± 4.5^**^ | 5/5 | 66.9 ± 6.3 |

Rhythmic mouse exhibits 24-h rhythmicity by cosinar analysis.

(a) Mouse numbers exhibited rhythmicity/total mouse number

(b) Same data in Fig.1. (c) Same data in Fig.5

* p<0.05, ** p<0.01 vs. No-Ex (intact No-Ex) evaluated using the one-way ANOVA test with Tukey *post*-hoc test.

# p<0.05, ## p<0.01 vs. No-Ex (intact No-Ex) evaluated using the Kruskal-Wallis test with Dunn *post*-hoc test.

$$ p<0.01 vs. SCNx-No-Ex evaluated using the one-way ANOVA test with Tukey *post*-hoc test.

+ p<0.05, ++ p<0.01 vs. intact No-Ex, & p<0.05, && p<0.01 vs. SCNx-No-Ex evaluated using Fisher’s exact test.

Figs. S1, S3, S8, and S10 are represented for supplementary figures.

**Supplementary Table S2. Cosinor analysis of daily rhythms of mRNA expression and of serum corticosterone shown in** Supplementary Figure S8.

| Gene | Tissue | No-Ex | T-Ex | W-Ex |
| --- | --- | --- | --- | --- |
|  |  | Acrophase (hr) | Phase shift vs. No-Ex (hr) | Phase shift vs. No-Ex (hr) |
| *Per2* | kidney | 17.0 | -1.6 | -0.4 |
|  | liver | 13.8 | -3.4 | -3.2 |
|  | sub gla | 16.6 | -2.0 | -0.4 |
|  | lung | 13.0 | -4.0 | -4.0 |
|  | gastrocnemius | 17.4 | -2.0 | -2.0 |
|  | adrenal | 14.6 | -3.4 | -3.8 |
| *Per1* | kidney | 16.6 | -1.2 | ±0 |
|  | liver | 13.4 | -3.2 | -4.4 |
|  | sub gla | 14.6 | -0.4 | -1.2 |
|  | lung | 10.6 | -1.2 | -1.2 |
|  | gastrocnemius | 15.4 | ±0 | +1.2 |
|  | adrenal | 9.8 | +0.4 | +0.4 |
| *Bmal1* | kidney | 20.2 | -3.2 | -2.4 |
|  | liver | 20.6 | -1.4 | -1.6 |
|  | sub gla | 21.8 | -1.6 | -2.0 |
|  | lung | 21.8 | -3.2 | -2.4 |
|  | gastrocnemius | 18.2 | -0.8 | -0.8 |
|  | adrenal | 20.6 | -1.6 | -1.6 |
| *Rev-erbα* | kidney | 9.0 | -13.2 | -12.8 |
|  | liver | 5 | -1.6 | -2.2 |
|  | sub gla | 9.0 | -4.0 | -4.8 |
|  | lung | 5.0 | -1.0 | -1.6 |
|  | gastrocnemius | 5.0 | -6.0 | -5.6 |
|  | adrenal | 5.0 | -3.6 | -2.8 |
| *StAR* | adrenal | 16.6 | ±0 | -0.2 |
| Corticosterone | Serum | 11.0 | -2 | -2 |

Acrophase was calculated by cosinor analysis, and phase differences between No-Ex and T-Ex/W-Ex were calculated for each clock gene, *StAR* gene, and corticosterone levels. Plus and minus values exhibit delay and advance from values of control (No-Ex), respectively.

Supplementary Table S3. Primer sequences for RT-PCR analysis.

| *Gapdh* | 5′-tggtgaaggtcggtgtgaac-3′ |
| --- | --- |
|  | 5′-aatgaaggggtcgttgatgg-3′ |
| *Per1* | 5′-caagtggcaatgagtccaacg-3′ |
|  | 5′-cgaagtttgagctcccgaagtg-3′ |
| *Per2* | 5′-tgtgtgcttacacgggtgtccta-3′ |
|  | 5′-acgtttggtttgcgcatgaa-3′ |
| *Bmal1* | 5′-ccacctcagagccattgataca-3′ |
|  | 5′-gagcaggtttagttccactttgtct-3′ |
| *Rev-erbα* | 5′-cttccgtgacctttctcagc-3′ |
|  | 5′-cagctcctcctcggtaagtg-3′ |
| *StAR* | 5′-gaaacaccttgcccacatct-3′ |
|  | 5′-ttgggcatactcaacaacca-3′ |
